# ﻿Life cycle and description of the immature stages of a terrestrial firefly endemic to Mexico: *Photinusextensus* Gorham (Coleoptera, Lampyridae)

**DOI:** 10.3897/zookeys.1104.80624

**Published:** 2022-06-07

**Authors:** Martín L. Zurita-García, Daniel Edwin Domínguez-León, Viridiana Vega-Badillo, Mireya González-Ramírez, Ishwari Giovanni Gutiérrez-Carranza, Geovanni M. Rodríguez-Mirón, Sara López-Pérez, Paulina Cifuentes-Ruiz, Miriam Aquino-Romero, Santiago Zaragoza-Caballero

**Affiliations:** 1 Departamento de Zoología, Instituto de Biología, Universidad Nacional Autónoma de México, Apartado Postal 70-153, 04510, Mexico City, Mexico Universidad Nacional Autónoma de México Mexico City Mexico; 2 Posgrado en Ciencias Biológicas, Universidad Nacional Autónoma de México, Edificio D, 1° Piso. Circuito de Posgrados, Ciudad Universitaria, 04510, Mexico City, Mexico Universidad Nacional Autónoma de México Mexico City Mexico; 3 Colección Coleopterológica, Museo de Zoología, Facultad de Estudios Superiores Zaragoza, Universidad Nacional Autónoma de México, Av. Guelatao 66, Col. Ejército de Oriente, Alcaldía Iztapalapa, 09230, Mexico City, Mexico Universidad Nacional Autónoma de México Mexico City Mexico

**Keywords:** Bionomics, egg, larva, Photinini, pupa, redescription

## Abstract

The life cycle, morphology, and bionomy of *Photinusextensus* Gorham, 1881, an endemic species of Mexico, are described. Redescriptions of adults (male and female) are also presented. Larvae were reared to the adult stage from eggs laid by females collected at the El Pedregal de San Ángel Ecological Reserve, south of Mexico City. The activity period of adults of *P.extensus* begins at the end of July and finishes by the end of August. Females lay between 3 and 198 eggs. Larvae hatch from the eggs after a period of 11 to 71 days, undergo 6 larval instars and a pupal stage in an annual cycle. Morphological characters of the sixth larval instar of *P.extensus* are compared with those of several other genera assigned to the tribe Photinini. Knowledge of the natural history of firefly larvae is relevant since most species do not feed as adults and therefore depend on resources acquired during the larval stage.

## ﻿Introduction

Fireflies belong to the family Lampyridae Rafinesque, 1815, and show a wide-ranging phenotypic and ecological diversity (Riley et al. 2021). Currently, there are more than 2400 described species with a worldwide distribution (Martin et al. 2019; Ferreira et al. 2020; Zaragoza-Caballero et al. 2020; Riley et al. 2021; Silveira et al. 2022). The highest species diversity is found in the Neotropical region (Costa 2000). Fireflies include nonluminous and luminous adults, luminous larvae and the females of some taxa are flightless (Branham 2010; Lewis et al. 2020). They inhabit wetlands, grasslands, forests, agricultural fields and urban parks (Lewis et al. 2020). Many fireflies are strongly associated with particular habitats and vegetation types (Faust 2017).

Firefly species can be either diurnally or nocturnally active. Diurnal species generally do not have light organs as adults and rely on pheromonal and visual cues (Ohba 2004; Branham 2010). Luminous species are nocturnal or crepuscular, with bioluminescent signals produced from photic organs of various shapes and sizes located on abdominal ventrites. These visual signals are typically used in sexual signaling to communicate species identity and facilitate pair formation (Branham 2010).

Firefly larvae can be aquatic, semiaquatic or terrestrial and can be found along the margins of streams and ponds as well as in leaf litter or rotten logs (Branham 2010). All known larvae are luminous and emit glows of varying duration. They are predatory on snails, earthworms and other soft-bodied prey (Lewis et al. 2020). The adults of most species do not feed and therefore rely on resources gathered during the larval stages (Lloyd 2002; Vaz et al. 2020). Multiple genera endemic to the Neotropical region have no larval or pupal descriptions. The few species that have been studied have different life histories (Vaz et al. 2020).

Currently, only the morphology of a small percentage of lampyrid larvae, at the generic or specific levels, of the approximately 144 genera and 2400 species, is known (Archangelsky 2010; Madruga and Branham 2020; Vaz et al. 2020; Zaragoza-Caballero et al. 2020; Riley et al. 2021; Silveira et al. 2022). There are a few studies describing the immature stages in the tribe Photinini. However, most of them have poorly detailed descriptions, composed only by the last larval stage (Riley et al. 2021). Besides, no tools to compare microstructures have been implemented. Of the 29 genera belonging to Photinini (Martin et al. 2019; Zaragoza-Caballero et al. 2020), there are detailed descriptions only for some species of *Pyractonema* Solier in Gay, 1849, *Pyropyga*, Motschulsky, 1852 *Lucidina* Gorham, 1883, *Lucidota* Laporte, 1833, *Phosphaenus* Laporte 1833 (Bugnion 1929; Beutel 1995; Branham and Archangelsky 2000; Archangelsky and Branham 2001; Archangelsky 2010, Kawashima 2017; Novák 2018b).

*Photinus* Laporte, 1833 is the most diverse genus of the subfamily Lampyrinae with more than 300 described species (McDermott 1964; Zaragoza-Caballero et al. 2020). Members of this genus live in a variety of habitats (from tropical dry forests to temperate and tropical montane cloud forests). Species range from the United States to Argentina. One species, recently collected in Spain, was described as new (Zaragoza-Caballero and Viñolas 2018) but then synonymized with the South American species *Photinussignaticollis* (Blanchard, 1846) (Koken et al. 2022).

In the past 20 years the number of known *Photinus* species has increased due to the description of new species from Mexico (Zaragoza-Caballero 2000, 2005, 2007, 2015, 2017; Zaragoza-Caballero et al. 2020). This fact contrasts with the lack of knowledge of the larval stages and the natural history of these organisms. Until now, no study has accurately documented the life cycle duration of a *Photinus* species. This lack of knowledge is the result of the difficulty of collecting mated females, as well as challenges associated with rearing larvae under laboratory conditions. Moreover, some species are known to spend several years in the larval stage, and the time fireflies need to complete their life cycle depends on the geographical region and the availability of larval food (Buschman 2017).

As for most genera in the family Lampyridae, *Photinus* larvae are poorly studied. Buschman (2017) observed that the first instars of this genus are smaller compared to those of *Photuris* Dejean, 1833, but no descriptions are provided. Most research documenting the natural history of *Photinus* focuses on the adult stage. These studies include courtship in males (flash communication) (Wing 1991; Viviani 2001; Faust and Weston 2009; Faust 2010), nuptial gifts (Lewis et al. 2004a, 2004b), the utilization of ejaculate-derived proteins to nourish developing oocytes in certain species (Demary 2005) and the size of signal detection and emission organs (López-Palafox et al. 2020).

This paper documents the life cycle and larval morphology of a *Photinus* species for the first time. *Photinusextensus* Gorham, 1881 is an endemic species of Mexico. Its known distribution includes the state of Chiapas, Hidalgo, Mexico, Morelos and Mexico City (Zaragoza-Caballero et al. 2020). A reduced population of this firefly was also found in El Pedregal de San Ángel Ecological Reserve, South of Mexico City. The life cycle of *P.extensus* is herein presented, with descriptions of larval instar 6. Some characteristics of instars 1 and 3 are mentioned; the adult was described as well.

## ﻿Methods

### ﻿Collection

**Figure 1.**
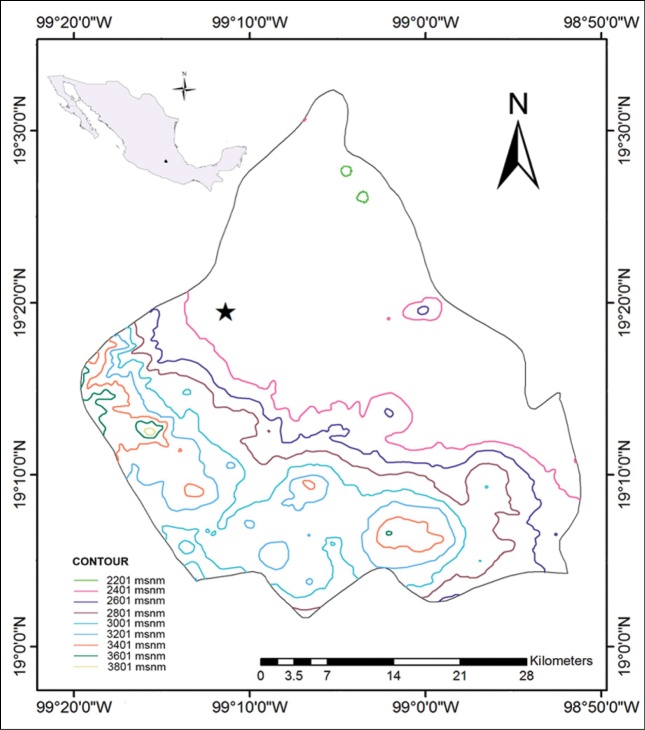
Map of Mexico City. Location of the study area where adults of *P.extensus* were collected (indicated with a star).

Adults of *P.extensus* were collected in the buffer zone of the El Pedregal de San Ángel Ecological Reserve (19°19'28.82"N, 99°11'20.95"W); this zone is between the core zone and the urban area of Mexico City, it is totally in the territory of the Universidad Nacional Autónoma de México. El Pedregal de San Ángel Ecological Reserve is located at the Southeast of Mexico City in the central Campus of the (**UNAM**) (Fig. 1). This community developed on a basaltic lava substrate approximately 1,670 years ago (Lot and Camarena 2009); the type of vegetation corresponds to a xerophytic shrub (Rzedowski 1978). The climate is sub-humid tempered with summer rains and an annual average precipitation of 833 mm; the annual average temperature is 15.5 °C (Orozco-Segovia et al. 2009). Fourteen adult females and 70 adult males of *P.extensus* were collected on August the 3^rd^, 8^th^, and 10^th^ of 2018, between 19:30 and 21 h. Adult specimens were located by their bioluminescence in the undergrowth, where the dominant plant species is *Pittocaulonpraecox* (Cav.) H. Rob. & Brettel (Asterales: Asteraceae). Other collections were made at the same site between June and July 2019, and five larvae were obtained. These developed into two female pupae and three males. Larvae, pupae, and adults were fixed in 70% ethanol for their preservation.

### ﻿Rearing in the laboratory

To observe the reproductive activity of *P.extensus*, adult specimens collected were divided into 14 groups consisting of five males and one female were placed in an 8 × 15 cm plastic container; peat moss substrate was added to simulate their natural environment. After oviposition, eggs were placed over a gauze patch in a 50 × 100 mm Petri dish, and moistened every 48 hours with an antimycotic solution based on Nistatine diluted in water (1/10). After eclosion, larvae werepartitioned into groups of five in separate Petri dishes (5×10 mm). To avoid dehydration, a filter paper layer was added and moistened every two days. This filter paper was replaced every week. Starting with the fourth larval instar, each larva was placed in a separate Petri dish (5×10 mm), with half of the dish covered with the filter paper, and the other half filled with sterilized dry sawdust. Following previous studies (Archangelsky and Branham 2001; Archangelsky 2010), larvae were fed small pieces of earthworm *Eiseniafetida* (Savigny, 1826) (Haplotaxida: Lumbricidae). Dead or partially consumed prey were removed from the Petri dishes every two days to keep containers clean. Pupae were maintained in Petri dishes (5×10 mm) at room temperature with sterilized dry sawdust until they completed their development. Specimens representing the different larval instars, pupae, and adults, were preserved in 70% ethanol for reference and subsequent study.

### ﻿Morphological study

**Figure 2.**
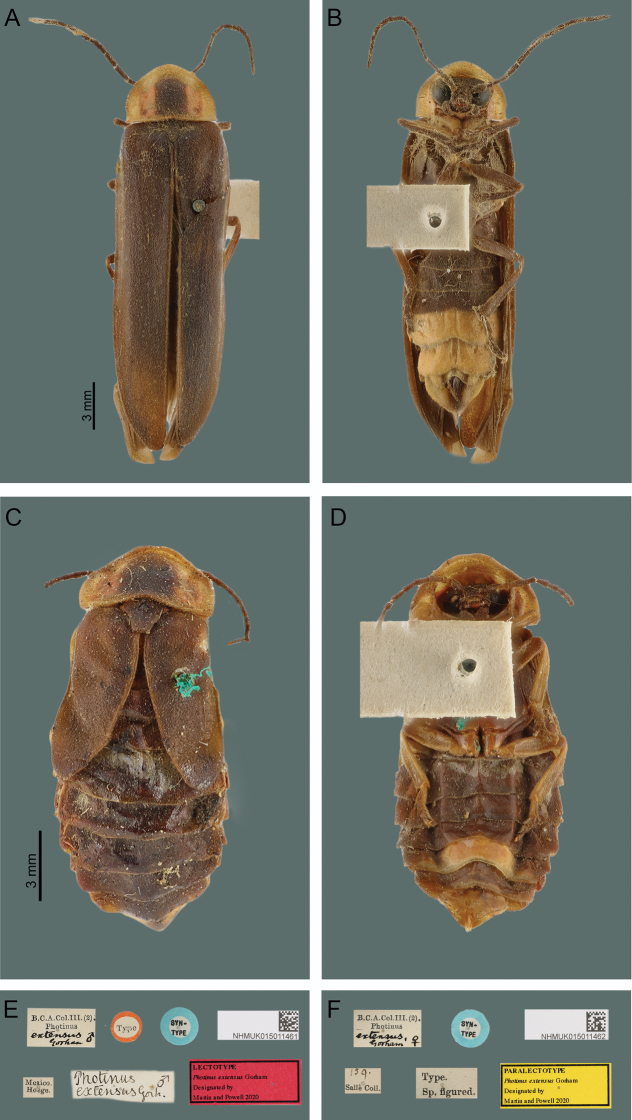
*Photinusextensus* Gorham, type specimens NHM-London **A** general habitus of the adult male in dorsal view **B** ventral view **C** general habitus of the adult female in dorsal view **D** ventral view **E** male labels (lectotype) **F** female labels (paralectotype). Photos: Keita Matsumoto.

Adults were identified using original descriptions, literature (Zaragoza-Caballero et al. 2000), and by comparison with photographs of type specimens (Natural History Museum of London, **BMNH**) (Fig. 2A–F) and specimens identified by experts deposited at the Colección Nacional de Insectos (**CNIN**), Instituto de Biología, UNAM, terminology of internal genitalia of females followed Silveira et al. (2022). Juvenile stages and adults of *P.extensus* were examined under a Zeiss stereoscopic microscope (Discovery V8) with a 1× objective lens coupled with 16× eyepieces. Larval heads of each instar were separated from the body and immersed in 10% KOH solution, the mouthparts were dissected under a stereoscopic microscope and placed in glycerin on slides for observation. Description of the distribution of the setae was made for the last instar larva following Branham and Archangelsky (2000) and Archangelsky (2010). Pygopodial structure for the last instar larva was interpreted using Fu et al. (2012). Redescription of the adult of *P.extensus* was made based on collected material. For the morphological description of larval instars, we followed the terminology of Novák (2018a) and Fu et al. (2012). A table 1 was made to compare morphological larval characters of Photinini (*Pyractonema*, *Pyropyga*, *Lucidota*, *Lucidina*, *Phosphaenus* and *Photinus*) (Branham and Archangelsky 2000; Archangelsky and Branham 2001; Archangelsky 2010; Novák 2018b), after Archangelsky (2010) and Kawashima (2017).

**Table 1.** Morphological larval characters of known Photinini genera (after Archangelsky 2010, Kawashima 2017 and Novák 2018b).

Character	*Pyractonemanigripennis* Solier	*Pyropyganigricans* (Say)	*Lucidotaatra* Olivier	*Lucidinaaccensa* Gorham	*Phosphaenushemipterus* (Goeze)	*Photinusextensus* Gorham
Body shape	Narrow, parallel 5.7–6.1× longer than narrow	Narrow, parallel	Narrow, parallel	Wide, suboval 4.2× longer than narrow	Oblong and narrow ~4.5× longer than narrow	Narrow, parallel ~5× longer than narrow
Ratio: body length/thoracic length	2.8–3.1	3.4–3.7	3.4–3.7	2.6	3.14	2.9
Cephalic capsule	Short and wide, retractable into the thorax	Short and wide, retractable into the thorax	Short and wide, retractable into the thorax	Subquadrate, moderately flattened dorso-ventrally.	Rectangular, retractable into the thorax	Short and wide, retractable into the thorax
Antennae	Wide, partially retracted into the head	Wide, partially retracted into the head	Wide, partially retracted into the head	Long and thin, completely retracted into the head	Long and thin, partially retracted into the head	Long and thin, partially retracted into the head
Opening of the mandible channel	At the exterior margin, subapical	At the exterior margin, subapical	At the exterior margin, subapical	At the exterior margin, subapical	At the exterior margin, subapical	At the exterior margin, subapical
Number of retinacula of the mandible	1	1	2	4	1	1
Maxillar palp	Three palpomere	Three palpomere	Three palpomere	Two palpomere	Tew palpomere	Four palpomere
Shape of pronotum	subcircular	subcircular	subcircular	subcircular	subcircular	suboval
Shape of mesonotum	sub oval	sub oval	sub oval	trapezoidal	sub oval	rectangular
Shape of metanotum	sub oval	sub oval	sub oval	rectangular	sub oval	rectangular
Thorax color	Dark with three pale, longitudinal, and subparallel lines	Dark with three pale, longitudinal and subparallel lines	Dark with three pale, longitudinal and subparallel lines	Almost blackish brown, lateral and hind margins more or less paler than the ground Subrectangular	dorssally dark reddish-brown, ventrally pink/ochre/light brown with darker plates on laterotergites and sternum	Dark, with pale stripes
Shape of the abdominal tergites	Subrectangular, except VII–IX subsquared	Suboval	Suboval, posterior margin of tergites V–VIII straight	Subrectangular	I–VII, IX subrectangular, VIII suboval	Subrectangular
Abdomen color	With a clear line on each side. Dark segments VII–IX.	Dark with pale lateral areas (segments I–VIII)	Dark with three pale longitudinal and subparallel lines, at the inner interior margin	Almost blackish brown, lateral and hind margins more or lesspaler than the ground I–VI, segments VII–X pale yellowish to milky white	pink/ochre/light brown	Pale with a little pink stripe in the second third in segments I–V, the rest of them pale

Images were taken with an AxiocamMRC5 camera attached to a Zeiss Axio Zoom V16 microscope with an objective lens Plan NeoFluar Z, 1×10.25 FWD 56 at the Laboratorio de Microscopía y Fotografía de la Biodiversidad II, Instituto de Biología, UNAM. Larvae were examined and imaged with a Hitachi SU1015 scanning electron microscope at the Laboratorio de Microscopía y Fotografía de la Biodiversidad I, Instituto de Biología, UNAM.

## ﻿Results

### ﻿ Photinus extensus

Taxon classificationAnimaliaColeopteraLampyridae

Gorham, 1881

73B0A7FE-746C-5424-B3CB-E35C150B1C7D

**Figure 3.**
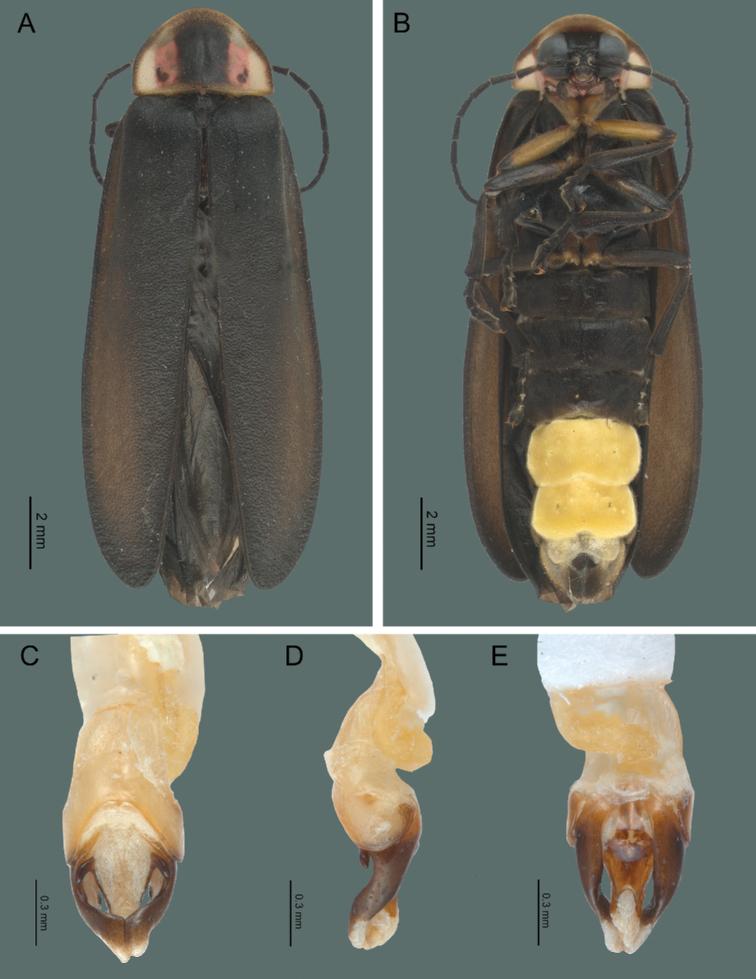
*Photinusextensus* Gorham **A** general habitus of the adult male in dorsal view **B** ventral view; Aedeagus: **C** dorsal view **D** lateral view **E** ventral view.

**Figure 4.**
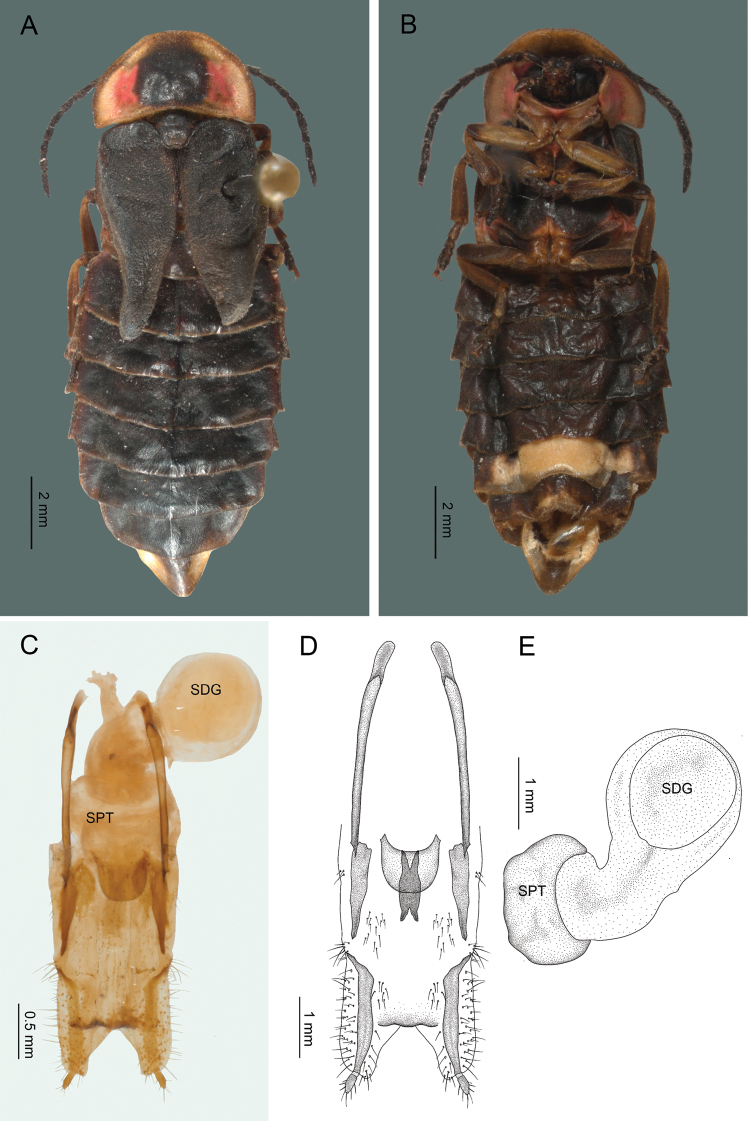
*Photinusextensus* Gorham **A** general habitus of the adult female in dorsal view **B** ventral view **C** internal genitalia **D** ovipositor dorsal view **E** internal anatomy of the reproductive tract: spermatophore digesting (SDG) and spermateca (SPT).

Figs 3A–E , 4A–E [Adults]

#### Redescription. Adult male

**(Fig. 3A, B) (n = 70).** Length of body 16.1–20.3 mm; width 3.7–4.2 mm. Body brownish, except the pronotal disk with a central black spot and two red spots at the sides; protrochanters, coxae, seventh and eighth ventrites yellowish; light organ in sternites 5–6 and 7^th^ sternite with diminished light spots.

***Head*.** Interocular space flat, almost parallel, shagreen-like integument, brilliant and pilose; frons vertical, interantennal distance (0.17–0.22 mm; 0.2 ± 0.01 mm) slightly wider than the antennal fossae (0.23–0.32 mm; 0.28 ± 0.04 mm); eyes finely faceted, semispherical, prominent, longer (1.1–1.18 mm; 1.17 ± 0.03 mm) than wide (0.62–0.98 mm; 0.9 ± 0.08 mm); antennae filiform, long (5.7–7.11 mm; 7.0 ± 0.11 mm), one-and-a half times longer than pronotum, extending beyond the posterior coxae, scape claviform reaching a length of (0.69–0.98 mm; 0.79 ± 0.10 mm), as long as the next two antennomeres together, the second short (0.19–0.79 mm; 0.28 ± 0.05 mm), the third to the tenth (0.54–0.71 mm; 0.66 ± 0.07 mm), the eleventh reaches (0.51–0.73 mm; 0.77 ± 0.03 mm); frontoclypeal suture membranous, almost straight; clypeus trapezoidal, anterior margin concave, with setae along the margin; mandibles falcate, robust, with setae on the external base; maxillar palpomere ogival and robust, labial palpomere securiform.

***Thorax*.** Pronotum wider (3.95–4.4 mm; 4.04 ± 0.08 mm) than long (2.8–3.4 mm; 3.01 ± 0.27 mm), semicircular, with a longitudinal groove indistinct in the basal half, anterior margin rounded, posterior sinuate, posterior angles straight, sides narrowly explanate, with irregular glandular pores at the front and ordered on the posterior and lateral margins, surface brilliant, abundant pilosity, decumbent; scutellum spatulate, with the posterior margin rounded, surface brilliant, punctate and decumbent pilosity; long elytra, parallel, four and a half times longer (12–13.5 mm; 12.6 ± 0.57 mm) than wide (2.4–2.8 mm; 2.58 ± 0.19 mm), surface rugose, opaque, with two types of pilosity, one relatively long and erect, the other small and procumbent; mesothoracic respiratory spiracles not tubular; long legs, pro, meso and metalegs similar to each other, femurs fusiform, tibiae channeled, a little dilated at the apex, external margin crenulate, two symmetric tibial spurs present in pro, meso and meta legs, tarsomeres laterally compressed, first metatarsomere longer (0.6–0.76 mm; 0.73 ± 0.03 mm) than the next two metatarsomeres together (0.51–0.68 mm; 0.63 ± 0.08 mm), fourth bifid, covering the fifth, claws simple.

***Abdomen*.** Sternites 5–6 longer than the preceding, with stigmatiform pores, posterior margin of sternite six cleaved, the seventh concave, the eighth ojival; posterior margin of pygidium convex; aedeagus short, robust, with symmetrical basal piece (0.67–0.7 mm; 0.68 ± 0.05 mm), as long as lateral lobes (0.66–0.7 mm; 0.67 ± 0.45 mm), with posterior margin concave, lateral lobes apically acute and convergent, median lobe cylindrical, with dorsal part membranous and ventral part with sclerosed base and apical half membranous, dorso-basal excrescences as long, oblique lobes, median orifice apical, lateral lobes narrowing towards the apex, apex blunt and wide (Fig. 3C, D, E).

#### Adult female

**(Fig. 4A, B) (n = 14).** Length: 11.1–20.3 mm; width: 3.2–6.5 mm. Body brownish, except the pronotal disk with a central black spot and two red spots at the sides; procoxae, protrochanters, meso-coxae, meta-coxae, seventh and eighth sternites yellowish; light organ in fifth sternite.

***Head*.** Interocular space flat, more or less parallel, shagreen integument, brilliant and pilose, frons vertical, interantennal distance (0.16–0.37 mm; 0.25 ± 0.10 mm) wider than antennal fossae (0.16–0.37 mm; 0.44 ± 0.16 mm) ; eyes small, finely faceted, semispherical, longer (0.65–0.94 mm; 0.67 ± 0.25 mm) than wide (0.45–0.76 mm; 0.54 ± 0.15 mm), antennae filiform, short (4.16–5.52 mm; 5.23 ± 0.96 mm), as long as the length of pronotum, without extending beyond the posterior margin of metasternum; scape reaching a length of (0.57–0.64 mm; 0.58 ± 0.34 mm), longer than the two next antennomeres together, the second short (0.2–0.39 mm; 0.37 ± 0.06 mm), from the third to the tenth (0.35–0.58 mm; 0.44 ± 0.07 mm), the eleventh reaches (0.54–0.69 mm; 0.68 ± 0.05 mm); frontoclypeal suture membranous, almost straight; clypeus trapezoidal, anterior margin concave, with setae along the margin; mandibles falcate, robust with setae on the external base; maxillar palpomere ogival and robust, labial palpomere securiform.

***Thorax*.** Pronotum wider (3.27–4.97 mm; 4.21 ± 0.86 mm) than long (2.21–2.6 mm; 2.2 ± 0.2 mm), semicircular, with a longitudinal groove indistinct on the basal half, anterior margin rounded, posterior margin straight, posterior angles straight, sides narrowly explanate, with glandular pores irregular at the front and ordered on the posterior and lateral margins, surface brilliant, pilosity abundant, decumbent, scutellum spatulate, with the posterior margin rounded, surface brilliant, punctate and pilosity decumbent; elytra short, without covering the abdomen, two-and-a-half times longer (4.62–6.5 mm; 5.2 ± 0.62 mm) than wide (1.91–2.71 mm; 2.22 ± 0.42 mm), surface rugose, opaque, pilosity decumbent; divergent in the median margin, epipleura reduced, mesothoracic respiratory spiracles not tubular; legs similar to each other; tibiae and femurs flat, fusiform, tibiae channeled, a little dilated at the apex, external margin crenulate, tarsomeres laterally compressed, first metatarsomere (0.41–0.64 mm; 0.62 ± 0.22 mm) slightly longer than the next two together (0.46–0.58 mm; 0.53 ± 0.06 mm), the fourth bifid, covering part of the fifth, claws simple.

***Abdomen*.** Sternites 5–6 longer than the preceding, without stigmatiform pores, posterior margin of sternite six almost straight, the seventh cleaved, the eighth with a notched; posterior margin of pygidium convex. Internal genitalia with a short and rounded spermatophore-digesting gland, longer than spermatheca, bursa copulatrix with an elongated and weakly sclerotized plate. Ovipositor with valvifers free, two-and-a-half times longer (2.41–3.10 mm; 2.75 ± 0.48 mm) than coxites (1–1.12 mm; 1.06 ± 0.08 mm); coxites divergent posteriorly; styli minute, sclerotized; proctigerplate short with rounded posterior margin, well-sclerotized (Fig. 4C, D, E).

**Description of pre-imaginal stages.**

#### Egg (Fig. 5A, B).

**Figure 5.**
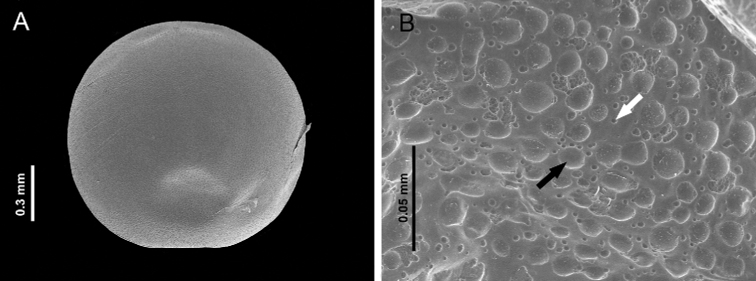
**A** Egg of *Photinusextensus* Gorham **B** surface with concavities that differ in size and shape (4–10 µm), evenly distributed, some present in aggregations.

Semispherical shape, whitish, with a diameter of approximately 290–300 µm (Fig. 5A). Surface with concavities that differ in size and shape (4–10 µm), evenly distributed, some present in aggregations (Fig. 5B). As time elapsed, the surface of the eggs became more transparent, allowing the observation of the larvae before hatching.

#### Sixth instar larva (Figs 6A, B, 7A–H, 8A–G, 9A–F).

**Description.** Elongate, tapering body, dorso-ventrally flattened, length 12.27–18.18 mm; integument of granular appearance; tergites from protergum to abdominal segment IX divided by sagittal line in dorsal view. Tergites with two lateral pale stripes that run throughout the body to the VIII segment, more sclerotized than the sternites, with clearly visible setae on the posterior margin of tergites VII to X; the last tergum completely dark except the lateral margins paler; anterior margin of the first head segment with two fossae (sensorial or glandular) paler and bigger than the rest of the punctations of the segment. Membranous pleura except for a dark sclerotized area around the spiracles, without apparent setae. The ventral surface is flexible due to the intersegmental membranes. Mesothoracic and abdominal pleural areas of segments I–VIII with bilabiate spiracles.

**Figure 6.**
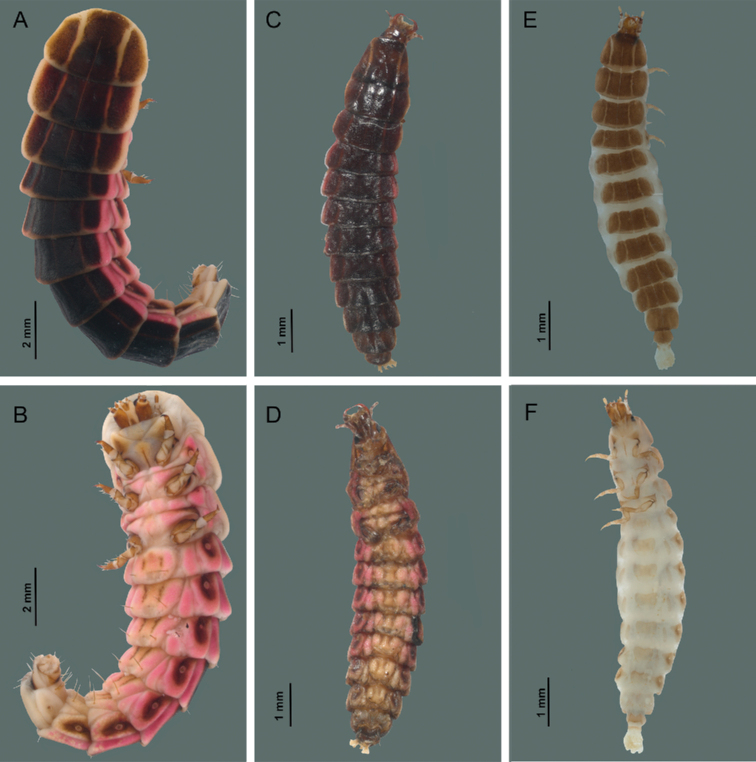
*Photinusextensus* Gorham **A** instar 6 in dorsal view **B** instar 6 in ventral view **C** instar 3 in dorsal view **D** instar 3 in ventral view **E** instar 1 in dorsal view **F** instar 1 in ventral view.

**Figure 7.**
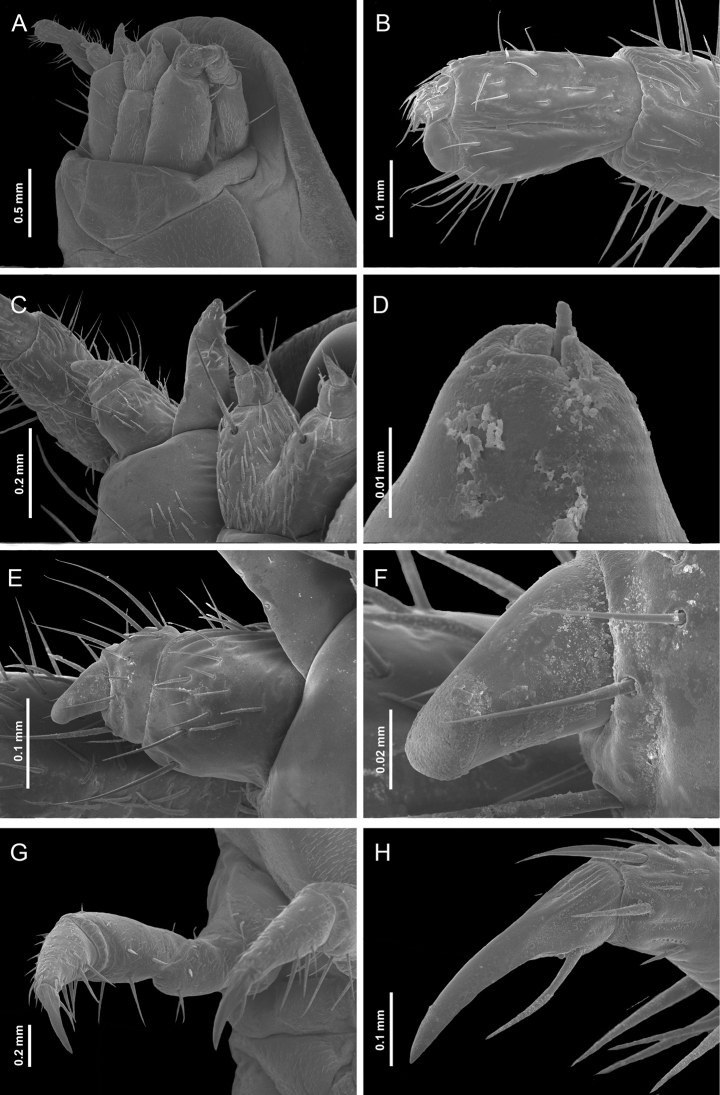
Sixth instar of *Photinusextensus* Gorham **A** SEM image of the head retracted and anterior part of prothorax in lateroanterior view **B** antennae **C** prementum, maxillary palpus and galea (lateroventral) **D** distal part of galea **E, F** maxillary palpi in ventrolateral view **G** right metathoracic leg in ventrolateral view **H** pretarsus.

**Figure 8.**
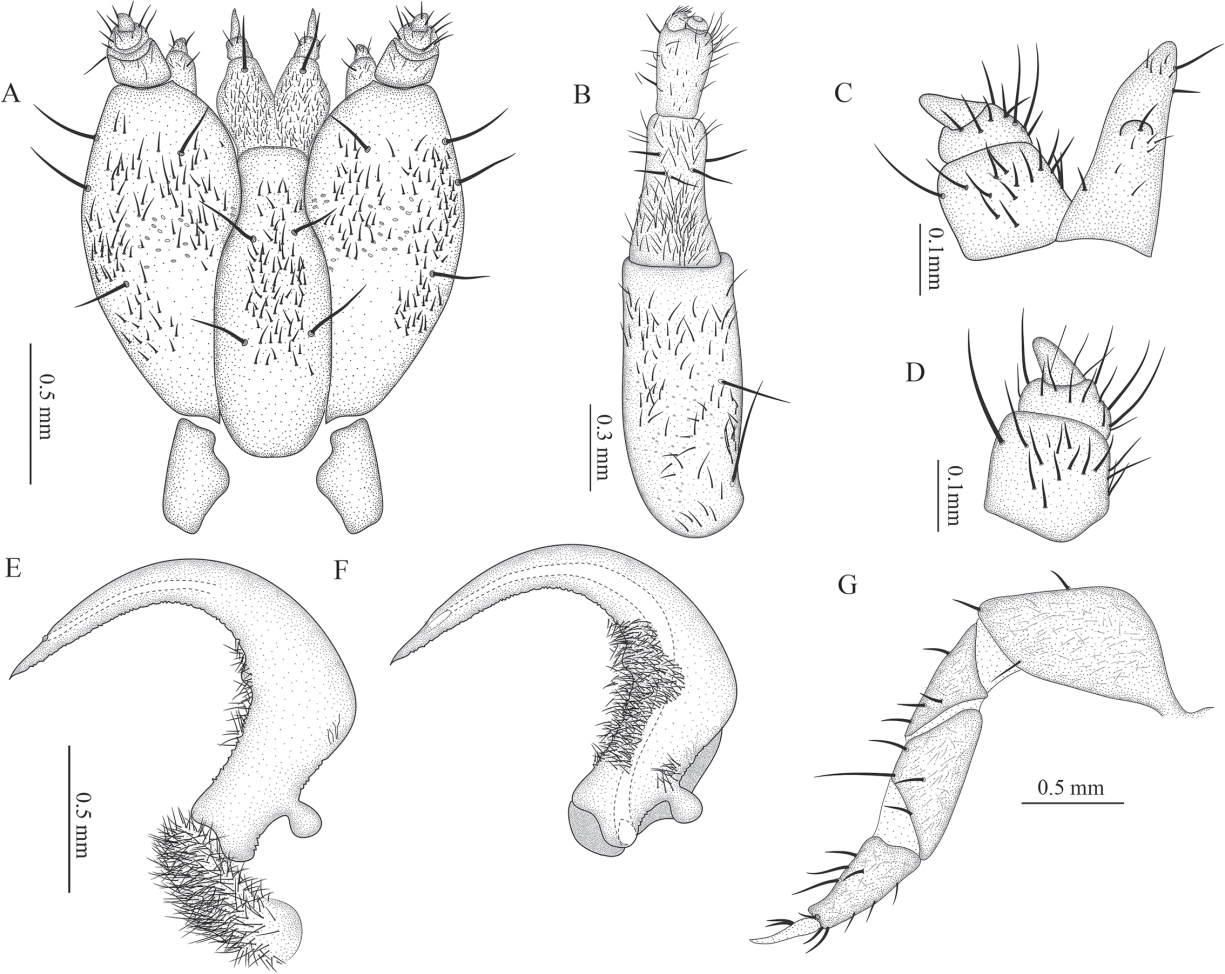
*Photinusextensus* Gorham, head structures of sixth instar larva **A** head capsule ventral view **B** right antenna, dorsal view **C** maxillary palpus and galea, ventral view **D** maxillary palpus, ventral view **E** mandible dorsal view **F** mandible ventral view and **G** leg dorsal view.

***Head capsule*.** Prognathous; slightly visible when retracted into prothorax due to the transparency of the protergum; extensible neck membrane covered in extremely short spines forms a two-layer envelope around the head; partially retractable within the prothorax; completely sclerotized, small, wider (0.88–1.54 mm; 1.2± 0.27 mm) than long (0.68–1.09 mm; 0.92 ± 0.17 mm), flat, sides almost parallel; stemmata on each side, with an almost transparent spot located posteriorly to the stemmata; clypeus and labrum fused forming the clypeo-labrum covering base of the mandibles in dorsal view; maxillae and labium connate forming maxillolabial complex covering most of the ventral cephalic area; epicranial suture dark, U-shaped, with a very short epicranial stem, frontal arms V-shaped (Figs 7A, 8A). Epipharynx formed by two oval plates, without setae, that project centrally beyond the anterior margin of the head. Hypopharynx with short setation.

***Antenna*.** Trimerous, located on the distal margin of the epicranial plate; partially retractable into the antennal socket; three-segmented, basal antennomere and second antennomere (0.42–0.55 mm; 0.48 ± 0.05 mm) elongated, and a third segment (the flagellum) short (0.24–0.31 mm; 0.26 ± 0.03 mm); adjacent sensorial cone present; basal antennomere with two long setae in the anterior mid, almost entirely covered by moderately dense, second antennomere with long setae close to apex and entirely covered by dense smaller finer setae, third antennomere with long setae from base to apex, with short setae on the anterior margin (Fig. 7B, 8B).

***Maxilla*.** Consisting of five parts, attached to lateral margins labium forming a maxillo-labial complex. Cardo elongate, irregular shape, with four setae in ventral surface, on long setae in posterior margin. Stipes elongated, ventrally covered with erect setae, with three long stout setae placed radially on the ventral apical region. Galea present, with two segments, the first longer and stouter than the apical, which is triangular (Fig. 7C, D). Lacinia covered with brush of long setae on outer lateral margin. Maxillae with three-segmented palpi, basal segment long (0.57–0.71 mm; 0.66 ± 0.06 mm) covered by setae in mid-region, segment II wider (0.30–0.40 mm; 0.35 ± 0.04 mm) than long (0.12–0.16 mm; 0.14 ± 0.01 mm); apical segment cylindrical (0.10–0.14 mm; 0.12 ± 0.01 mm) with numerous setae from base to mid region; (Figs 7E, F, 8C, D).

***Labium*.** Closely attached to maxilla, formed by prementum, mentum and postmentum. Prementum heart-shaped, surface covered with numerous short setae and two long setae close apex; labial palpi with two segments, basal palp subquadrate with few setae in mid-region, distal palp conical without setae; mentum with one pair of setae on anterior third and one pair of setae on posterior third; postmentum elongate, slightly sclerotized at the medial base, laterally united by membranes to the cardines; with a setae on each side near the base.

***Mandible*.** Symmetrical, falcate, strongly sclerotized, with an internal channel opening subapically on outer edge. Penicillus well-developed. Retinaculum short and rounded, present only as a blunt protuberance on basal third of the mandible. Densely covered by fine setae on the external margins basely, basal half on inner margin of mandible covered with a brush of stout setae, being longest on the retinaculous protuberance (8E, F); mesal margin serrate.

***Thorax*.** Protergum wider (2.43–3.81 mm; 3.14 ± 0.57 mm) than long (1.54–2.59 mm; 2.11 ± 0.43 mm), subsemicircular, wider posteriorly, rounded at posterolateral corners, covering the retracted head. Meso- and metatergum subrectangular three times wider than long, delimited by a pleural suture elongate barely evident from the laterotergites. Lateral areas of meso and metathorax scarcely sclerotized, composed of two laterotergites, the anterior with a well-developed spiracle on the mesothorax. Episterna extending from the anterior part to the lateral part of the coxae; epimeron forming a little sclerotized stripe, parallel to the coxae.

***Legs*.** Pentamerous, the first pair of forelegs slightly shorter than the second and third. Coxae short (0.76–1.16 mm; 0.98 ± 0.18 mm), cylindric, widely separated at the base, decumbent; coxal-trochanteric membrane reaching about 1/3 of the coxal length. Trochanters pentagonal, joining the femur obliquely (0.51–0.82 mm; 0.71 ± 0.13 mm). Femur narrow and cylindrical in lateral view. Tibiotarsus narrowing distally with stout setae. All legs with a double row of long setae in the inner margin, numerous short setae in the outer margin: pretarsus claw-like with two setae at base (Figs 7G, H, 8G).

**Figure 9.**
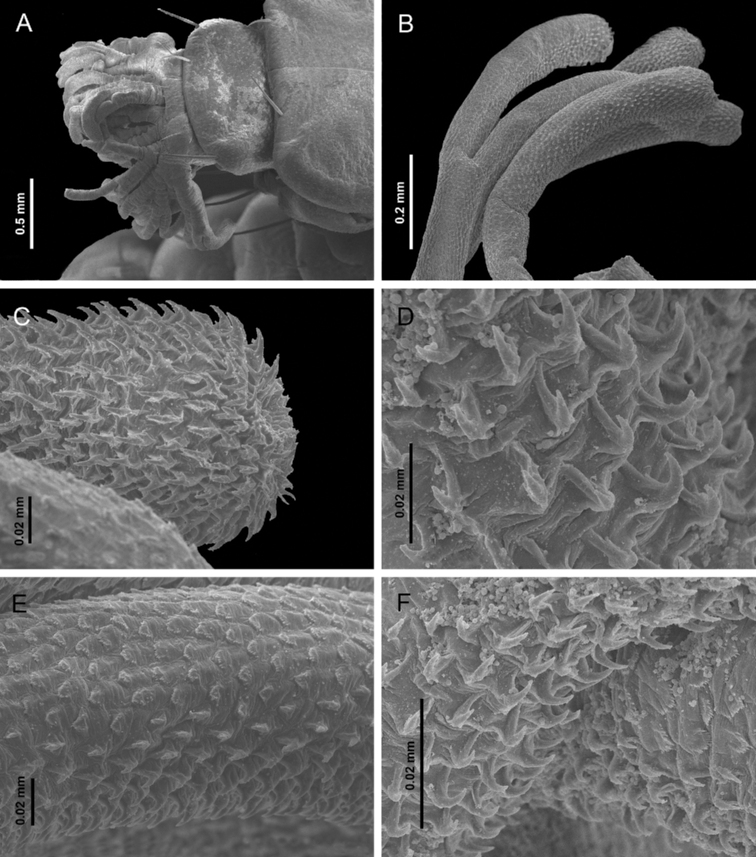
Sixth instar of *Photinusextensus* Gorham **A, B** SEM image of pygopodium in general view and detailed **C–F** detail of the surface of pygopodium.

***Abdomen*.** Tergites III–IX of similar length (0.86–1.36 mm; 1.09 ± 0.17 mm), width almost constant; segments I–VIII (or I–VII) wider than long, bearing a pair of long stout setae posterolaterally, with laterotergites at each side, with sclerotized plates containing the spiracles; ventral area of segments I–VIII with sternal areas almost squared, slightly pigmented, sternites with two long setae in mid-region; sternal medial area margined by laterosternites – sometimes pigmented, elongate, narrow, and paired, delimited by laterotergites dorsally, and ventrally by a medial sternal plate; ventral area of segment IX with a simple plate, without area differentiation; light organ present and segment VIII indistinct; abdomen ending with a series of eversible filaments (pygopodia) bifurcate at the apex, at least 30 pygopodia arise from 12 basal stalks which may branch more than once (the dorso and ventrolateral stalks branch into three); densely packed recurved hooks occur on the ventrolateral surface of each exerted pygopod and completely covering at apex, with toothed scales on the dorsolateral surface only on anterior half. (Fig. 9A–G).

#### Third instar larva (Fig. 6C, D).

**Description.** Similar to sixth instar. Length 11.45–13.78 mm. ***Head capsule*.** Wider (0.82–0.96 mm; 0.92± 0.05 mm) than long (0.57–0.85 mm; 0.71 ± 0.12 mm). ***Antenna*.** Basal antennomere and second antennomere (0.28–0.35 mm; 0.32 ± 0.02 mm), and a third segment (the flagellum) (0.15–0.21 mm; 0.18 ± 0.02 mm). ***Maxilla*.** Maxillae with three-segmented palpi, basal segment long (0.34–0.54 mm; 0.43 ± 0.07 mm) covered by setae in mid region, segment II wider (0.30–0.43 mm; 0.36 ± 0.06 mm) than long (0.12–0.17 mm; 0.14 ± 0.02 mm); apical segment cylindrical (0.10–0.13 mm; 0.11 ± 0.01 mm). ***Thorax*.** Protergum wider (1.36–1.53 mm; 1.4 ± 0.06 mm) than long (1.18–1.68 mm; 1.3 ± 0.20 mm), trapezoidal. ***Legs*.** Coxae short (0.77–0.97 mm; 0.86 ± 0.08 mm), femur obliquely (0.43–0.60 mm; 0.52 ± 0.07 mm). ***Abdomen*.** Tergites III–IX (0.68–0.85 mm; 0.77 ± 0.045 mm) ventral area of segments I–VIII with sternal areas almost squared, slightly pigmented, sternites with two long setae in mid-region.

#### First instar larva (Fig. 6E, F).

**Description.** Similar to sixth instar. Length 3.72–8.80 mm; after hatching, the first instar body does not appear sclerotized to the degree found in later instars. ***Head capsule*.** Wider (0.48–0.72 mm; 0.6± 0.10 mm) than long (0.24–0.51 mm; 0.38 ± 0.12 mm), s (Figs 6A and 7A). ***Antenna*.** Basal antennomere and second antennomere (0.12–0.28 mm; 0.19 ± 0.06 mm) elongated, and a third segment (the flagellum) short (0.08–0.17 mm; 0.12 ± 0.03 mm). ***Maxilla*.** Maxillae with three-segmented palpi; basal segment long 0.24–0.47 mm; 0.31 ± 0.08 mm) and well-defined, segment II wider (0.12–0.17 mm; 0.15 ± 0.01 mm) than long (0.05–0.07 mm; 0.06 ± 0.007 mm); apical segment (0.03–0.047 mm; 0.037 ± 0.007 mm). ***Labium*.** Postmentum elongate, slightly sclerotized at the medial base, laterally united by membranes to the cardines; with a setae on each side near the base.

***Thorax*.** Protergum wider (0.73–1.47 mm; 0.94 ± 0.21 mm) than long (0.51–0.75 mm; 0.53 ± 0.09 mm). ***Legs*.** Coxae short (0.44–0.65 mm; 0.53 ± 0.08 mm), femur obliquely (0.21–0.34 mm; 0.27 ± 0.05 mm). ***Abdomen*.** Tergites III–IX (0.31–1.2 mm; 0.74 ± 0.28 mm).

#### Pupa, male (Fig. 10A, B).

Length 17–23 mm; width 6–7 mm. Body elongate, curved, ventrally concave, pale yellowish (sternites I–VIII slightly pigmented at the ends).

**Figure 10.**
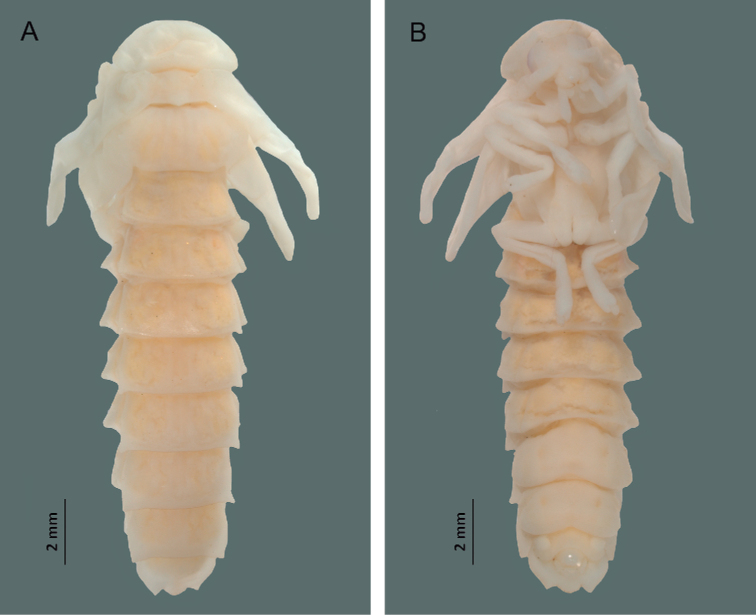
*Photinusextensus* Gorham **A** pupa in dorsal view **B** pupa in ventral view.

***Head*.** Totally covered by the pronotum in dorsal view. Large eyes, located at the sides of the head; antennae in front of the eyes, nearer the frontal center, mouthparts visible in ventral view.

***Thorax*.** Pronotum wider than long, semicircular, totally covering the head. Meso and metanotum shorter, subrectangular, bearing the elytra sidewards. All pairs of legs free, visible in ventral view. Spiracles present in the pleural areas of mesothorax.

***Abdomen*.** Abdominal segments subrectangular, wider than long, spiracles present on abdominal pleural areas of segments I–VIII. Light organ on sternites V–VI.

#### Life cycle (Fig. 11).

In their natural habitat, adults of *P.extensus* are active from early July, when the first males can be observed. Bioluminescent activity begins at dusk, at approximately 20:00 h, and diminishes considerably an hour later. Male flight does not exceed 2 m in height. Females are brachypterous and perch in the undergrowth approximately 50 cm from the ground. Males flash every 4.5 seconds, flying in an arc when illuminated. When males detect a female, they wait for an intense flash as a response, which is brief. The flash intervals are of 10 to 20 seconds. Males react by flying lower and towards the female. Groups of 3 to 5 males commonly compete with each other to get the female first, to mate with her. Two types of competition were observed among males: 1) a mating ball: four or more males cover the copulating pair and try to dislodge the copulating male to gain access to the female, and 2) males using their pronotum as a lever to pry a copulating male from the female. In the laboratory, copulation was observed to last from between 2 to more than 4 hours.

**Figure 11.**
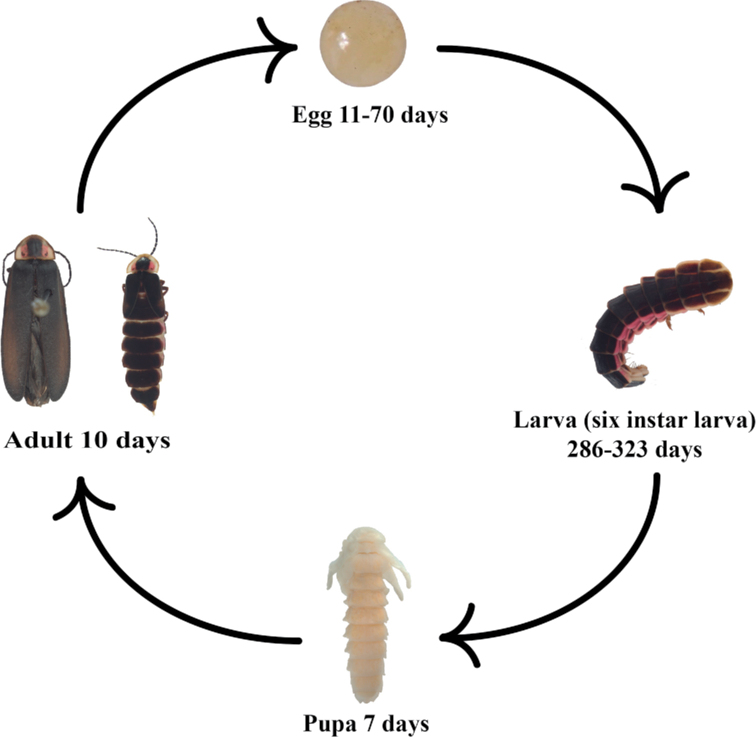
Life cycle of *Photinusextensus* Gorham.

During oviposition, females bend their abdomen and place the apical part of it on the substrate. Eggs are laid superficially or buried, randomly distributed, individually, or in groups (up to aggregates of 50). The number of eggs deposited by each female varied from three to 198. Eggs emit a faint bioluminescence since they are oviposited, which is only perceptible to the human eye in complete darkness. In total, 956 eggs from 13 females were obtained.

Under lab condition *P.extensus* completed its development in approximately 12 months, from oviposition to imago. The egg stage under laboratory conditions had a duration of 11 to 70 days, with mortality of n = 144 eggs (15%).

*Photinusextensus* undergoes six similar larval instars that differ in both size and color (Fig. 4). Cannibalism among larvae during rearing was not observed. The only food larvae consumed was the earthworms provided. There was no synchronization among larvae during the progression of larval instars, which started at the end of August until the beginning of July. In captive conditions, the process of ecdysis from one stage to the other varied among individuals. The first larval instar had a duration of 14 to 153 days, where mortality was 60% (n = 491) among the eggs that hatched. The second larval instar had a duration of 14 to 172 days, with mortality of 61% (n = 199). The third larval instar had a duration of 15 to 140 days; mortality was 71% (n = 87). The fourth larval instar had a duration of 17 to 140 days, with a mortality of 52% (n = 20). The fifth larval instar had a duration of 24 to 192 days (n = 18) and the last larval instar had a duration of 53 days (n = 8). Pupation had a duration of 7 days in July, and according to the observations in the field, pupae were found under pyroclastic rocks.

## ﻿Discussion

*Photinusextensus* has six instars; they are very similar and only differ in size, color and the degree of the body sclerotized and presence of setae. Instar III differs from I and VI by a trapezoidal pronotum (Fig. 5) and an exposed head. The periods of time of the instar I–V are variable in different specimens, ranging from 14 and 192 days; the last instar period in different specimens is constant, approximately 50 days; the pupa is completely developed in seven days.

Frequently, the identification and description of larvae in the tribe Photinini is based on the characters present in the final larval instar, mostly body shape, color pattern, head capsule features, and the morphology of mouthparts (Archangelsky 2010). There are both similarities and differences between larvae belonging to the tribe Photinini, including *P.extensus* (see Table 1). The larval character suite found in *P.extensus* is most similar to those found in *Pyractonema*, *Lucidota*, and *Pyropyga*. The major exception to this is the number of segments in the maxillary palp. Although this last character is shared with the genus *Phosphaneus*, it differs in the opening of the mandible channel. *P.extensus* differs from *Lucidina* in the number of segments in the maxillary palp, and the number of retinacula of the mandible. There are some patterns among the larvae described in Photinini; the shape of the body is narrow and parallel and the pronotal shape is semicircular or semioval and the opening of the mandible channel is at the inner margin subapical.

The distribution of the setae is different between genera (Table 2), chaetotaxy will be useful in Lampyridae as a tool to distinguish between immatures, also as a source of informative phylogenetics characters (Ballantyne et al. 2019; Riley et al. 2021; Vaz et al. 2021). However, the few studies on the morphology of immatures in Lampyridae and the lack of knowledge of chaetotaxy hinder comparison between fireflies and makes the elaboration of more detailed hypotheses very difficult.

**Table 2.** Chaetotaxy larval characters.

	*Photinusextensus* Gorham	*Pyractonemanigripennis* Solier	*Lucidotaatra* Olivier
**Hypopharynx**	Margin covered by dense pubescence.	Surface covered by dense pubescence.	No information.
**Mandible**	With patches of dense pubescence in the basal part in ventral Margin covered by dense pubescence. With patches of dense pubescence in the basal part in ventral view. Mid-region covered by a single row of long setae.	With patches of dense pubescence in ventral view. Mid-region covered by only one row of setae.	Mid-region covered by only one row of long setae. One long seta close to the apex.
**Cardo**	With 4 long setae in ventral surface and one long seta in the posterior margin.	With 13–15 setae in ventral surface.	Without setae.
**Maxillary palpomeres**	Basal palp covered by setae in the mid region. Distal palp with setae from base to mid region.	Basal palp covered by setae. Palp II with some setae. Last palp without setae.	Basal palp with long setae from the mid region to the apex. Palp II with shorter setae. Distal palp without setae.
**Labial palpomeres**	Basal palp with few setae in mid region. Distal palp without setae.	Basal palp with some setae. Distal palp without setae.	Basal palp with few long setae. Distal palp with one long seta.
**Prementum**	Surface with many setae. Two long setae close to apex.	Dorsal and ventral surface with many setae.	With two basal regions of very fine setae, with longer setae on the palp segment.
**Submentum**	With two long setae in mid region.	With two long setae in the basal middle.	No information.
**Antennomeres**	Apex with two long setae. Antennomere III with long setae from base to apex.	All antennomeres covered by setae. Antennomere III with many short setae.	Basal antennomer with mid region covered by setae. Anterior region with longer setae. Antennomere II covered evenly by long setae. Antenomere III with short setae.
**Legs**	With a double row of long setae in the inner margin. Outer margin with many setae. Pretarsus with two setae at the base.	With a double row of long setae in the inner margin. Pretarsus with two setae at the base.	With a double row of long setae in the inner margin. Pretarsus with two setae at the base.
**Abdomen**	Sternites with two long setae in mid-region.	Sternites with two long setae in mid-region.	No information.

The larval characters have shown to be important to clarify the phylogenetic relationships as Archangelsky (2010) mentioned. However, it seems to be a difficult task to get a good dataset of larval characters of Lampyridae.

The life cycle, morphology, and behavior of the species of *Photinus* are similar. *Photinuscarolinus* Green, 1956, *P.ignitus* Fall, 1927, *P.marginellus* LeConte, 1852, *P.pyralis* (Linnaeus, 1767), *P.greeni* Lloyd, 1969, and *P.extensus* are the known species that produce spermatophores due to the prolonged time of copulation (Cratsley et al. 2003; South and Lewis 2012).

The length of the pupal stage varies slightly in *Photinus*. The pupal stage of *P.extensus* and *P.carolinus* has a duration of six days (Faust 2010). Nevertheless, the pupa of *P.carolinus* is present during May, while that of *P.extensus* is present in July. Another difference is that the pupae of *P.extensus* observed in the field were found under pyroclastic rocks, in contrast with *P.carolinus*, which has been reported to occur under leaf-litter, near rotten logs, or moss (Faust 2010). Until now, little information has come to light about the pupae in other genera of Photinini (Archangelsky and Branham 2001). In other genera like *Aspisoma* Laporte, 1833, of Cratomorphini, the pupal stage is similar, occurring during a short period between six and ten days (Costa et al. 1988; Archangelsky 2004).

## ﻿Conclusion

The complete life cycle of *Photinusextensus*, including descriptions of egg, larvae, and pupa, was documented for the first time. Larvae were reared in laboratory conditions to the adult stage from eggs. The six instar of *P.extensus* are very similar; they differ only in size and in the sclerotized degree.

Among Photinini larvae there are not many differences, differing in the number of segments in the maxillary palp and in the number of retinacula of the mandible; the shape of the body and pronotum, and the opening of the mandible channel follow a similar pattern. Life cycle information is essential to carry out protection and conservation actions for insects that are very sensitive to environmental changes, like fireflies. For example, the species that do not produce light are easily overlooked and the information about their life cycle is deficient. This results in “Data Deficient” categorization in evaluations of extinction risk. Thus, more studies are needed in which the life history, habitat associations, and microhabitat are detailed (Fallon et al. 2021). Also, it is necessary to understand that requirements of larvae are different to those of adults to have an integral vision in the actions of protection of the fireflies.

## Supplementary Material

XML Treatment for Photinus extensus
